# A Structural View of miRNA Biogenesis and Function

**DOI:** 10.3390/ncrna8010010

**Published:** 2022-01-18

**Authors:** Ana Lúcia Leitão, Francisco J. Enguita

**Affiliations:** 1 MEtRICs, Department of Sciences and Technology of Biomass, NOVA School of Science and Technology, FCT NOVA, Universidade NOVA de Lisboa, 2829-516 Caparica, Portugal; aldl@fct.unl.pt; 2 Instituto de Medicina Molecular João Lobo Antunes, Faculdade de Medicina, Universidade de Lisboa, Av. Prof. Egas Moniz, 1649-028 Lisboa, Portugal

**Keywords:** microRNA, biogenesis, structural biology, X-ray crystallography, cryo-electron microscopy, nuclease, Dicer, DGCR8, Drosha, Exportin-5, Argonaute, RISC

## Abstract

Micro-RNAs (miRNAs) are a class of non-coding RNAs (ncRNAs) that act as post-transcriptional regulators of gene expression. Since their discovery in 1993, they have been the subject of deep study due to their involvement in many important biological processes. Compared with other ncRNAs, miRNAs are generated from devoted transcriptional units which are processed by a specific set of endonucleases. The contribution of structural biology methods for understanding miRNA biogenesis and function has been essential for the dissection of their roles in cell biology and human disease. In this review, we summarize the application of structural biology for the characterization of the molecular players involved in miRNA biogenesis (processors and effectors), starting from the X-ray crystallography methods to the more recent cryo-electron microscopy protocols.

## 1. Introduction

In 1993, the research groups led by Victor Ambros and Gary Ruvkun published two side-by-side papers in the *Cell* journal, describing the regulatory effects exerted by a tiny RNA discovered in *Caenorhabditis elegans* [1,2]. According to the obtained experimental evidence from both groups, a small RNA named lin-4, and expressed following an heterochronic pattern, was characterized as a post-transcriptional regulator of the lin-14 gene. Interestingly, the initial results also showed that the regulatory action of lin-4 over lin-14 mRNA was exerted by a direct binding of the ncRNA to the 3′-UTR region of the coding transcript [1,2]. The term “microRNA” (miRNA) was only used years later by Tom Tuschl [3], Victor Ambros [4] and David Bartel [5] to designate this family of small post-transcriptional regulators. Almost 10 years after their discovery, miRNAs were already described in complex organisms including mammals and plants [6,7,8], and they were also related with some human diseases [9]. In the last decade, the importance of miRNAs in human pathophysiology has been revealed by specific studies that connected their regulatory actions to the onset, establishment, progression and prognosis of diverse conditions such as cancer [10,11], and cardiovascular [12,13] and metabolic diseases [14,15].

The molecular details of miRNA regulatory action were unveiled during the 2000′s decade. Interestingly, miRNA-mediated downregulation of gene expression was rapidly related with the phenomenon of RNA-mediated interference (RNAi), suggesting that both mechanisms could share common cellular effectors [16]. As a general rule, miRNAs act as negative post-transcriptional regulators of mRNA transcripts by a mechanism that involves a base pairing between the miRNA and the 3′-UTR of the mRNA and a protein complex (RNA-induced silencing complex, RISC) which is directed to the target by the miRNA–mRNA interaction [17]. Moreover, the miRNAs showed a distinctive biogenesis pathway, where specific transcriptional units are processed by a group of defined nucleases to generate the small regulatory RNA [18]. Equivalent biogenesis pathways are also observed in other short regulatory RNAs, such as PIWI-interacting RNAs (piRNAs) [19], but are not present in the biosynthesis of long non-coding RNAs (lncRNAs) [20] or circular RNAs (circRNAs) [21], which are dependent on the common cellular transcription and RNA maturation machineries.

**Figure 1:**
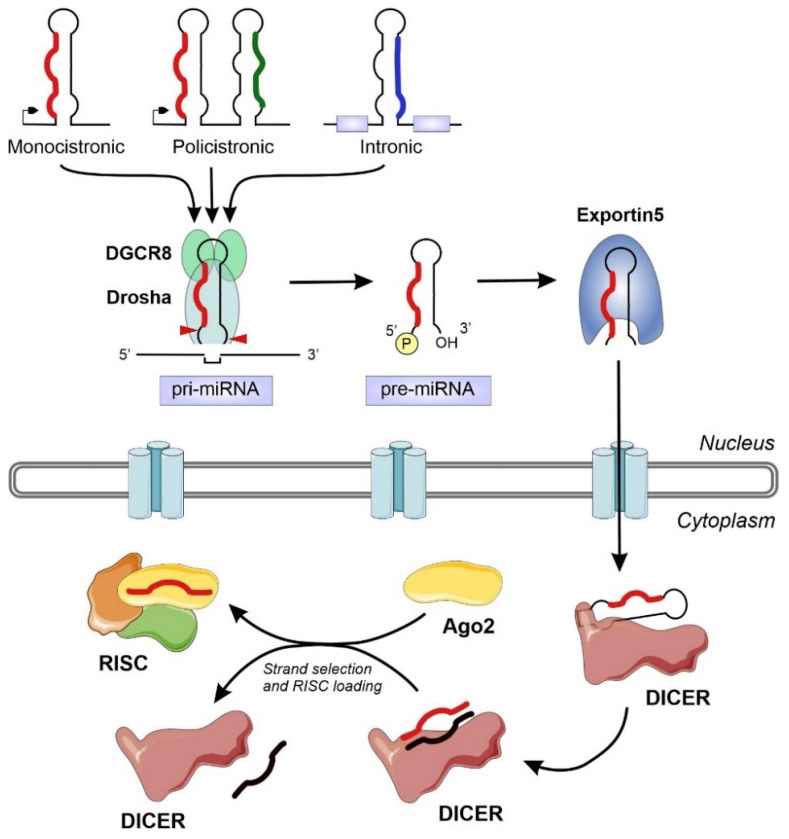
Schematic representation of the miRNA biogenesis pathway, including all the relevant enzymes and cellular compartments involved. The eukaryotic genomes contain transcriptional units that will generate miRNAs. These information units, that appear isolated or clustered, can be located in several genomic territories, including intergenic regions, coding and non-coding genes. After transcription, the typical hairpin-loop secondary structure present in primary miRNAs (pri-miRNAs) is recognized and excised by the microprocessor complex (composed by DGCR8 and Drosha). The generated precursor miRNAs (pre-miRNAs) will be exported to the cytoplasm and further processed by the Dicer nuclease to generate a dsRNA. The mature miRNA chain will be selected by Ago2 and engaged together into the RNA-induced silencing complex (RISC) to exert its regulatory action.

The miRNA biogenesis flows from the transcription of devoted information units by RNA polymerase II to the production of the final functional regulators by successive steps of maturation [18] (Figure 1). We can distinguish two different groups of enzymes involved in miRNA biogenesis: *processors*, integrated by RNA endonucleases that cleave the miRNA precursors in the nucleus (Drosha) and the cytoplasm (Dicer), and *effectors*, including the Argonaute family and all the proteins responsible for the post-transcriptional regulatory effect over the mRNA targets (RISC complex) [22]. The overall biogenesis pathway must be complemented with the transport system responsible for the export of miRNA precursors from the nucleus to the cytoplasm (Exportin-5) [23] and other helper proteins that modulate the whole process in specific steps, such as TRBP and DGCR8 [17,24]. The nuclear processors are exclusive for the biosynthesis of miRNAs, whereas the cytoplasmic branch of the biogenesis pathway can be shared with other regulatory mechanisms such as the RNA-mediated interference (RNAi) (Figure 1).

During the last decade, structural biology has benefit from the rapid development of new methods and from the adaptation and improvement of the already existing ones. This can be exemplified by the design and development of new X-ray detectors with improved sensitivity and range [25] or by the adaptation of new protocols for sample processing for cryo-electron microscopy (cryo-EM) analysis [26]. The structural characterization of the different players involved in miRNA biogenesis has rapidly evolved in parallel with the development of new techniques and methods. In this review, we analyzed the highlights of structural biology applied to the field of miRNA biogenesis, showing the evolution of this fascinating field from the first crystal structures of Argonaute proteins isolated from extremophilic bacteria to the more recent high-resolution cryo-EM structures of human nuclear and cytoplasmic miRNA processors.

## 2. Nuclear Microprocessor: Drosha and DGCR8

After transcription by RNApol II, the transcriptional units devoted to the production of miRNAs generate a primary miRNA (pri-miRNA). In all the cases, pri-miRNAs harbor an internal hairpin-loop structure that will be recognized and processed within the nucleus by a tandem of interacting proteins: the RNA-binding protein DGCR8 and the RNAse III nuclease Drosha [24,27]. Additional proteins such as the ERH splicing factor can also interact with the microprocessor Drosha-DGCR8 complex, modulating its catalytic activity and the biogenesis of miRNAs [28].

In humans, DGCR8 protein is encoded by a gene located in the DiGeorge syndrome chromosomal region (DGCR, chromosome 22q11.2) [29]. It consists of 14 exons spanning over 35 kb and produces a canonical transcript with an ORF of 2322 bp that encodes a protein of 773 amino acids. The human DGCR8 protein harbors two dsRNA-binding domains located in the C-terminal region of the protein (amino acids 512–576 and 620–684) [24]. The N-terminal domain contains long sections of intrinsically-disordered residues, which suggest the existence of flexible segments that could facilitate the interaction with functional partners [27]. DGCR8 protein acts as an essential partner of the Drosha RNAse by recognizing the apical region of the hairpin pre-miRNA and simultaneously behaving as an anchor for the Drosha enzyme that will process the dsRNA segment [30]. Isolated evidence also connected the DGCR8 protein with the heme group, where this cofactor has been described as a modulator of the oligomerization state of DGCR8 [31]. The heme-bound DGCR8 dimer seems to trimerize upon binding pri-miRNAs and is active in triggering pri-miRNA cleavage, whereas the heme-free monomer is much less active [31].

The inherent intrinsically disordered nature of DGCR8 has prevented its easy characterization by structural methods. In fact, the first report of a structural analysis of the DGCR8 protein deposited in the PDB database corresponds to still unpublished data. These data depict the structure determination of one of the dsRNA binding domains by solution NMR, obtained within the framework of the RIKEN structural genomics initiative in 2005 (PDB code: 1X47) (Table 1). Two years later, Sohn and coworkers solved the crystal structure of the DGCR8 core comprising the amino acids from 493–720 [32]. The structural data showed a well-defined tandem of dsRNA binding domains arranged by a pseudo two-fold symmetry axis connected to a terminal alpha helix. Additional experimental evidence also suggested the ability of the protein to recognize the pri-miRNA structure in two possible orientations, which could be related with the processing efficiency of the associated Drosha nuclease [32]. Further molecular details were obtained from two following structural studies characterizing the DGCR8 dimerization domain by X-ray crystallography in *Homo sapiens* [33] and *Xaenopus levis* [34].

**Figure 2:**
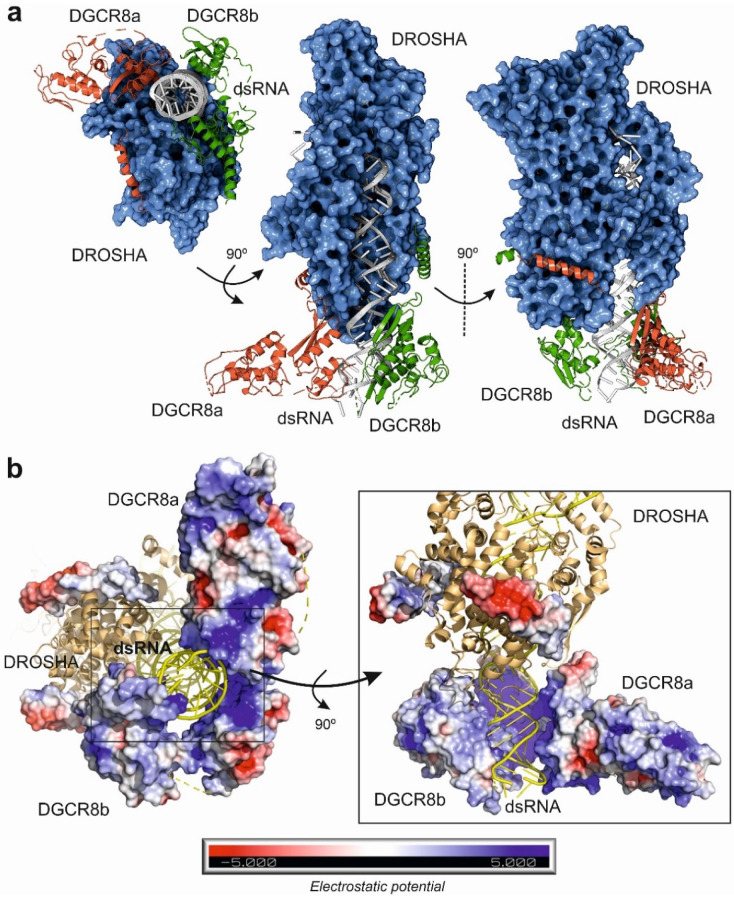
Different views of the human microprocessor complex structure composed by the Drosha endonuclease, two monomers of DGCR8 protein (a and b) and a synthetic pri-miRNA as determined by cryo-electron microscopy (PDB code: 6V5B) [37]. (**a**) Overview of the microprocessor complex structure, showing how the dimerized DGCR8 protein can recognize and bind to the basal segment of the pri-miRNA, acting as an anchor for the further binding of the catalytic RNAse Drosha. (**b**) Detailed view of the interaction between the pri-miRNA and the microprocessor complex, showing the electrostatic potential distribution over the surface of the DGCR8 dimer and the positively charged pocket that accommodates the base of the pri-miRNA substrate. Figure was prepared with 3D Protein Imager [39] and PyMOL [40].

In 2016, Kwon and coworkers solved the X-ray structure of human Drosha in complex with a small fragment of DGCR8 protein to a resolution of 3.2 Å [36]. These data were complemented by two additional studies by cryo-EM that characterized the structure of Drosha–DGCR8 in complex with a synthetic pri-miRNA [37,38] (Table 1). Drosha showed a complex fold comprising an apical PAZ-like domain, two dsRNA binding domains and a catalytical nuclease region which is connected to the core of the protein by a short amino acid stretch. Altogether, the domains form a deep cavity to receive the pri-miRNA hairpin loop (Figure 2a). The cryo-EM data also allowed to determine that the microprocessor core is formed by a closely packed DGCR8 dimer that interacts with the basal region of Drosha and the terminal pri-miRNA loop (Figure 2b). The functional requirement of DGCR8 dimerization for interaction with the dsRNA was clearly demonstrated by the presence of a positively charged tunnel that stabilizes the assembly of the catalytic microprocessor (Figure 2b). Interestingly, cryo-EM structures also showed how the catalytic interaction of Drosha with the pri-miRNA is ensured by hydrophobic interactions with the DGCR8 core. These results suggested that the specificity of cleavage by the microprocessor is dependent on the recognition of the apical region of the pri-miRNA by the DGCR8 dimer that will trigger the further assembly and catalysis of the Drosha RNAse [36,37,38]. The structural information derived from the high resolution cryo-EM structures of the microprocessor also suggests that the heme-binding region of DGCR8 is responsible for the recognition of the terminal loop of the pri-miRNA, whereas the RNA-binding domain of DGCR8 will be attached to the apical half of the stem. Once assembled, the distance between the terminal stem and the Drosha catalytic center will be around 35 nucleotides. The tandem DGCR8–Drosha structure assembled around the pri-miRNA stem look will provide stability of the pre-loading complex and specificity of the maturation reaction [37,38].

Despite the reliable and strong structural information about the nuclear microprocessor, the presence of structurally unfolded segments in DGCR8 and Drosha proteins suggest the existence of additional components of the complex that could act as modulators of the pri-miRNA processing reaction. Very recent evidence demonstrated the participation of an additional RNA-binding protein and splicing factor, ERH, in the function of the microprocessor [28]. In fact, by using a combination of structural and biochemical methods, ERH has been described as an interaction partner of DGCR8 and modulator of the microprocessor activity. Biochemical data suggested that ERH is an assistant protein which is important in the maturation of miRNA clusters [28].

## 3. Cytoplasmic Processors: Dicer

After the initial trimming by the Drosha/DGCR8 complex in the nucleus, the precursor miRNAs (pre-miRNAs) will be transported across the nuclear pores by the carrier protein Exportin-5 (Exp-5). The nuclear exporting machinery is completed by the small nuclear guanine triphosphatase (GTPase) Ran (RanGTP) [41]. Structure of the Exp-5/RanGTP in complex with a pre-miRNA was solved by X-ray diffraction [42]. The structural analysis of the pre-miRNA export system allowed to determine that Exp-5/RanGTP recognizes the 2-nucleotide 3′-overhang structure and the double-stranded stem of the pre-miRNA, shielding the pre-miRNA stem from degradation [42]. The interaction between the Exp-5 transport cage and the pre-miRNA is enhanced by electrostatic interactions between the inner Exp-5 cavity and the negatively charged pre-miRNA backbone [23,42]

Once in the cytoplasm, the pre-miRNA hairpin is processed again by the Dicer ribonuclease to generate a dsRNA that will be the source of the mature and functional miRNAs. Like Drosha, Dicer is a conserved bidentate type-III RNAse that was initially characterized as an important player of the RNAi pathway. In 2001, simultaneous publications from the laboratories of Gregory Hannon [43], Craig Melo [44] and Philip Zamore [45] described how Dicer was essential in the biogenesis of dsRNAs and in the subsequent interference process by using different models. Further studies characterized the precise mechanism used by Dicer to generate size-specific fragments from long dsRNAs and explained why the enzyme was an essential point to connect the RNAi and miRNA biogenesis pathways [46,47]. However, the first structural data of a Dicer enzyme were only available in 2006 when Jennifer Doudna’s research group published the crystal structure of a primitive Dicer enzyme from the human parasite *Giardia intestinalis* [48]. Despite the simplicity of the enzyme, several striking conclusions can be derived from this pioneer study: first, the structure of the enzyme showed a multidomain organization, harboring a PAZ-like domain for RNA binding [49] which was separated from the catalytic RNAse III domain by a connecting segment; second, the distance from the PAZ-like domain and the RNAse III domain was 65 Å, which is compatible with the length of the pre-miRNA substrate (around 23–25 dsRNA base pairs). All these observations allowed the authors to propose the term of “molecular ruler” to describe how Dicer enzyme is able to recognize pre-miRNAs and to generate dsRNAs of a precise length, determined by the molecular dimensions of the enzyme and the location of the functional domains [48,50].

**Figure 3:**
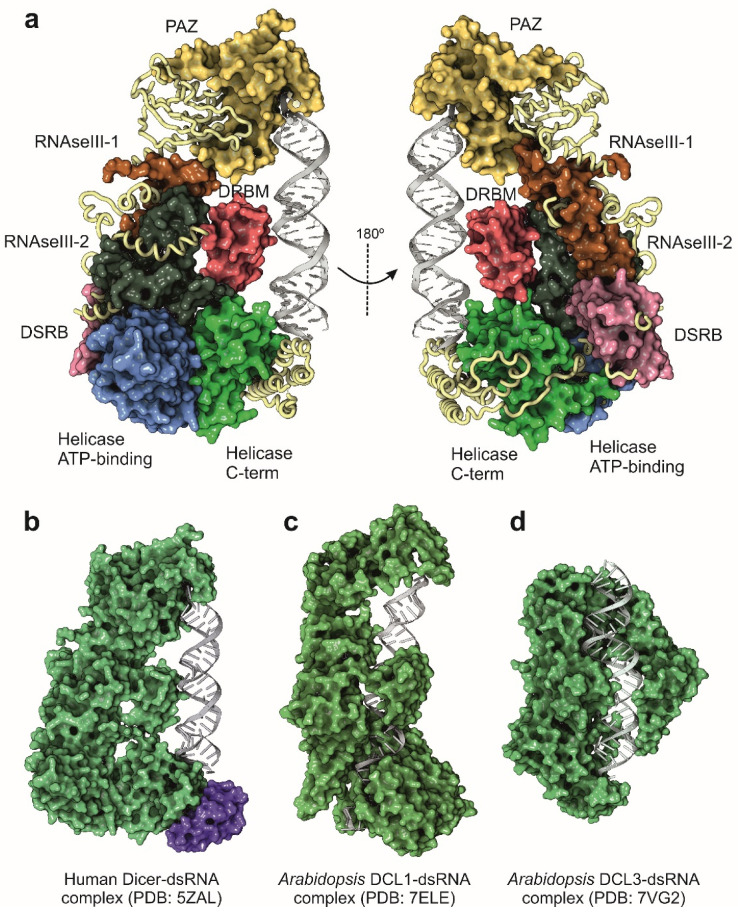
Structure of Dicer and Dicer-like proteins determined by cryo-EM. (**a**) Functional domains of human Dicer protein. (**b**) Structure of human Dicer in complex with the TRBP protein, a component of the RISC complex (magenta surface) and the pre-let-7 [51]. (**c**) Structure of the Dicer-like protein 1 (DCL1) from *A. thaliana* in complex with the pre-miR-166f [71]. (**d**) Structure of the Dicer-like protein 3 (DCL3) from *A. thaliana* in complex with a synthetic 40 mer dsRNA [72]. All the structures were rendered and represented with Protein Imager software [39].

During the following years after the publication of the structure of *G. intestinalis* Dicer, the attempts to characterize the full-length enzyme from higher eukaryotes by structural methods were unsuccessful. The only available source of information was based on the truncated structures of functional Dicer domains (Table 2). However, and thanks to the rapid improvement of cryo-EM methods, the structure of a full-length human Dicer in complex with a pre-miRNA substrate was determined in 2018 at 4.7 Å resolution [51]. Compared with the *Giardia* enzyme, human Dicer is more complex in its domain organization, and closely related with other Dicer enzymes from mammals and insects. Tertiary structure of human Dicer shows a L-shape arrangement that forms a pocket to accommodate the pre-miRNA (Figure 3a,b). The basal segment of the protein is composed by two helicase-like domains, very similar to those observed in the family of ATP-dependent DExD/H-box helicases [52]. The function of the helicase-like domain in human Dicer is not totally understood since the human protein does not depend on ATP for its catalytic activity [46]. Other Dicer proteins, such as the enzyme from *Drosophila,* also contain the helicase domain, but their enzymatic activities are dependent on the hydrolysis of ATP [53]. In consequence, the helicase domain in human Dicer could be probably related with the recruitment of additional partners such as TRBP but also with the modulation of the dicing activity [54,55]. The human protein also contains two dsRNA-binding domains, the DRBM domain that interacts with the dsRNA minor groove and the DSRB domain (also known as domain of unknown function, DUF), probably involved in the recognition and binding to the pre-miRNA (Figure 2a) [56]. The two catalytic RNAse III domains are located close to the cavity formed by the folding of the terminal PAZ domain. The PAZ domain (Piwi-Argonaute-Zwilli domain), also present in Drosha and Argonaute proteins, is necessary for the proper binding of the enzyme to the 3′-unpaired end of the pre-miRNA and appears to be widely conserved in dsRNA-binding enzymes [57].

Structural biology information combined with biochemical assays also allowed to dissect specific molecular details involved in the dicing activity and additional modulators of the pre-miRNA maturation [58]. For instance, the flexibility of a sliding bulge located within the catalytic RNAse III region of Dicer has been involved in the alternative processing mechanism observed in humans and higher eukaryotes for the generation of miRNA isoforms from the same pre-miRNA precursor [59]. Additional partners of Dicer, such as the mammalian TRBP, were early characterized as modulators of the RNAi mechanism and regulators of the processing of endogenous pre-miRNAs [55,60]. Structural studies by NMR combined with surface plasmon resonance were employed to characterize the RNA binding mechanism exhibit by TRBP [60] and to explain the modulatory effect of pre-miRNA maturation when interacting with Dicer (Figure 2b) [60].

**Table 2:** Representative PDB database entries describing full-length or domain structures of Dicer and other cytoplasmic processing enzymes for miRNA biogenesis.

PDB Code	Biomolecule	Species	Technique	Resolution	Year	Reference
2FFL	Apo-Dicer	*Giardia intestinalis*	X-ray	3.3 Å	2006	[48]
2QVW	Apo-Dicer twinned data	*Giardia intestinalis*	X-ray	3.0 Å	2007	[61]
2EB1	Dicer C-terminal domain	*Homo sapiens*	X-ray	2.0 Å	2007	[62]
3C4B	Dicer RNAseIIIb-dsRNAb domain	*Mus musculus*	X-ray	1.6 Å	2008	[63]
2KOU	Dicer-like protein	*Arabidopsis thaliana*	NMR	N/A	2010	[64]
3ADL	TRBP2	*Homo sapiens*	X-ray	2.2 Å	2010	[65]
3RV0	Dcr1 without C-terminal domain	*Vanderwaltozyma polyspora*	X-ray	2.2 Å	2011	[66]
2L6M	Dcr1 C-terminal dsRBD domain	*Schizosaccharomyces pombe*	NMR	N/A	2011	[67]
2LRS	Dicer-like 1 dsRBD domain	*Arabidopsis thaliana*	NMR	N/A	2012	[68]
4NGB	Dicer Platform-PAZ-connector	*Homo sapiens*	X-ray	2.2 Å	2014	[69]
4WYQ	Dicer-TRBP interface	*Homo sapiens*	X-ray	3.2 Å	2015	[55]
5F3P	Non-canonical Dicer protein	*Entaboeba histolytica*	X-ray	1.9 Å	2018	Unpublished
6BU9	Dicer-2 complexed with dsRNA	*Drosophila melanogaster*	Cryo-EM	6.8 Å	2018	[56]
5N8L	TRBP dsRBD siRNA complex	*Homo sapiens*	NMR	N/A	2018	[60]
5ZAL	Dicer complex with pre-miRNA	*Homo sapiens*	Cryo-EM	4.7 Å	2018	[51]
7DEY	Apo-Dicer	*Scheffersomyces stipitis*	X-ray	2.8 Å	2021	[70]
7ELE	DCL1 in complex with pre-miRNA	*Arabidopsis thaliana*	Cryo-EM	4.9 Å	2021	[71]
7VG2	DCL3 in complex with RNA	*Arabidopsis thaliana*	Cryo-EM	3.1 Å	2021	[72]

The characterization of human Dicer by structural methods supported the previous evidence obtained from the genome mining data regarding the evolutionary conservation of dsRNA processing enzymes [71,73,74]. Plants and simple eukaryotic genomes frequently harbor genes encoding for several Dicer-like proteins (DCL) that could be considered as an evolutionary reminiscence derived from the primitive antiviral defense mechanisms [75,76], whereas complex eukaryotes typically have a single Dicer-encoding gene [77,78].

Plants are particularly interesting for miRNA functional studies since they have an increased diversity in the structure and sequence of the miRNA genes, joined to the existence of different DCL protein isoforms [76,79]. Cryo-EM analysis has been recently employed for the characterization of the structure of two plant DCL enzymes (DCL1 and DCL3) in complex with synthetic dsRNAs (Figure 3c,d) [71,72]. Despite the existence of different DCL enzymes, miRNA maturation in plants is ensured only by the action of DCL1, which is able to combine the Drosha and Dicer activities in a single enzyme, recognizing pri-miRNAs and performing two sequential cleavage reactions to generate the mature miRNAs [80]. The structure of *A. thaliana* DCL1 determined by cryo-EM suggested that the PAZ domain together with the helicase domain are responsible for the plasticity of the catalytic activity of the enzyme. The helicase domain serves as a transfer module to perform the two sequential cleavage reactions that convert the pri-miRNA into a mature miRNA, whereas the PAZ domain would stabilize the protein–RNA complex during the enzymatic maturation of the precursors [71]. Interestingly, plants appear to have a different processing system for siRNAs with the involvement of several DCL isoenzymes. Moreover, in *A. thaliana*, a recent cryo-EM structure of DCL3 allowed to characterize an independent mechanism for the generation of siRNA from long dsRNA precursors [72]. The structures of DCL1 and DCL3 from *A. thaliana* (Figure 3c,d) differ in their domain configuration, being that the DCL3 isoenzyme is less complex and adaptative for the processing of long dsRNA molecules.

## 4. Cytoplasmic Effectors: Argonaute Proteins and RISC Complex

The post-transcriptional regulation of a miRNA over a cognate target is ensured by the recruitment of the mature miRNA into the RISC complex, which will be assembled around the target and guided by the base complementarity between the miRNA and the mRNA [17]. The assembly of an active RISC complex requires a transfer of the mature miRNA from the last processing step catalyzed by Dicer to the molecular effector [81]. The cytoplasmic RISC complex is a very dynamic assembly formed by different proteins, being AGO2, a member of the Argonaute family, the core regulatory element [82]. Argonaute (AGO) proteins are conserved RNA binding proteins that, in eukaryotic cells, couple the biogenesis of miRNAs and other small ncRNAs to their physiological actions [17]. AGO proteins are responsible for the loading of the dsRNA produced by Dicer into the RISC by a process that is dependent on the intervention of RNA chaperones and ATP hydrolysis [83] and the selection of the mature miRNA strand [84].

Initially characterized in plants as essential developmental regulators [85], AGO proteins were lately described in many complex organisms as general players of the RNA-mediated regulatory events namely RNAi and post-transcriptional modulation by miRNAs [86,87,88]. Interestingly, Argonaute-like proteins are also found in bacterial proteomes, suggesting that their role as modulators of RNA-based regulatory phenomena is widespread along evolution [88,89]. In bacteria, Argonaute proteins behave as site-specific endonucleases which are guided by short nucleic acid sequences to find their DNA or RNA targets [90,91,92], whereas in higher eukaryotes they are usually the core of RNA-mediated regulatory complexes involved in post-transcriptional regulation [83,93]. The diverse families, functions and roles of AGO proteins have been extensively reviewed elsewhere [87,94,95].

**Figure 4:**
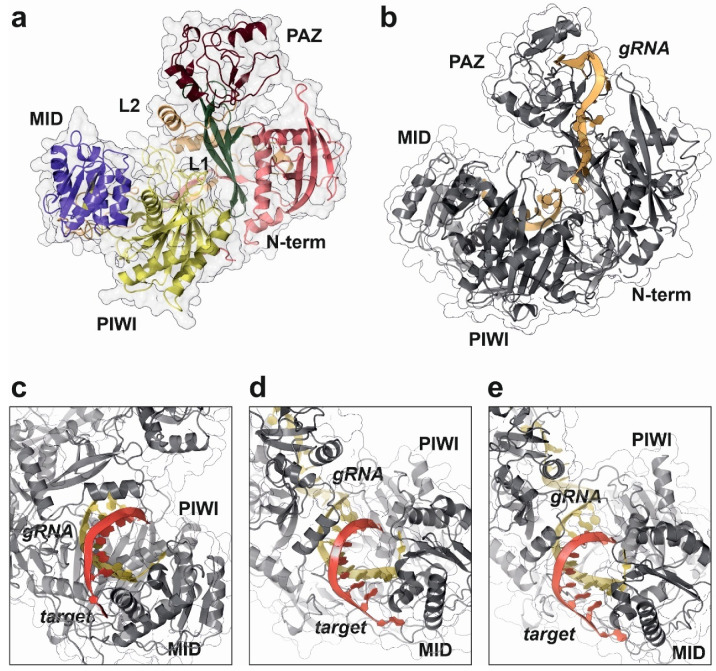
Structural details of human AGO2 protein complex with a synthetic guide RNA (gRNA) and cognate targets with different pairing specificities, as determined by X-ray crystallography experiments. This work proposed the “walking model” for target recognition and engagement by AGO2 driven by the dynamic base pairing of the miRNA seed sequence [109]. (**a**) Domain distribution in human AGO2 structure (PDB code: 7KI3). (**b**) Surface representation of human AGO2 in complex to a synthetic gRNA (yellow ribbon), showing the characteristic open pocket that accommodates the dsRNA hybrid during target recognition (PDB code: 4W5N). (**c**) Detailed view of the RNA-binding pocket in human AGO2, showing the target-guide dsRNA hybrid with a 2–7 seed pairing (PDB code: 4W5T). (**d**) Human AGO2 targeting dsRNA complex with a 2–8 seed pairing (PDB code: 4W5Q). (**e**) Human AGO2 structure complex with an RNA guide and a 2–9 seed pairing target (PDB code: 4W5O). All the figure panels were prepared with the Protein Imager software [39].

The first structural characterization of a full-length AGO protein was published in 2004 (Table 3). Song and coworkers used X-ray crystallography to solve the structure of an Argonaute protein isolated from *Pyrococcus furiosus* to 2.25 Å resolution and its apo form (PDB code: 1U04) [90]. Further studies published in the following year characterized the molecular mechanism used by an Argonaute-like protein from *Archaeoglobus fulgidus* to recognize and bind small regulatory RNAs (PDB code: 1YTU) [96]. Bacterial Argonaute proteins are nucleic acid-guide endonucleases, whose tertiary structure comprises four distinct domains (PIWI, PAZ, MID and N-terminal), arranged in an open shape that defines a central catalytic cavity. This tertiary structure characterized in the bacterial proteins is strongly conserved along evolution and can also be observed in the mammalian and human AGO counterparts (Figure 4a) [97].

Human genome encodes for four different AGO proteins, named from 1 to 4. Genes encoding AGO1, AGO3 and AGO4 are clustered together in a 300 kb region located in the small arm of chromosome 1, whereas AGO2 gene is in chromosome 8. AGO proteins are ubiquitously expressed across different tissues in humans and their specific functions are still a subject of study [98]. The different human AGO paralogs share a high degree of sequence and structural homology (Supplementary Material Figures S1 and S2). Initial evidences showed that only AGO2 retains the RNA-directed nuclease activity observed in the prokaryotic homologs, being involved in the induction of mRNA degradation in RNAi [99], whereas the remaining paralogs should be responsible for the translational repression modulated by endogenous small ncRNAs. In absence of AGO2, the RNAi and cleavage of the target transcript is inefficient when performed by targeting the coding sequence of an mRNA transcript (CDS). In AGO2-knockout cells, small RNAs targeting the 3′-UTR of mRNAs were also able to induce a post-transcriptional repression driven by the engagement of AGO1 and AGO3 proteins into the RISC complex [100]. Consequently, a competition among AGO paralogs is expected under physiological conditions; however, only AGO2 appears to be a catalytically active RNA endonuclease. This fact is supported by the observation that the different AGO proteins do not show binding preferences for any specific family of miRNAs [101].

**Table 3:** Representative PDB database entries describing full-length structures of Argonaute proteins and complexes with different nucleic acids.

PDB Code	Biomolecule	Species	Technique	Resolution	Year	Reference
1U04	Full length Argonaute	*Pyrococcus furiosus*	X-ray	2.2 Å	2004	[90]
1W9H	Apo form Piwi protein	*Archaeoglobus fulgidus*	X-ray	1.9 Å	2004	[102]
2BGG	siRNA/Piwi protein complex	*Archaeoglobus fulgidus*	X-ray	2.2 Å	2005	[103]
1YVU	Full length Argonaute	*Aquifex aeolicus*	X-ray	2.9 Å	2005	[104]
3DLB	RNA/Ago complex	*Thermus thermophilus*	X-ray	2.7 Å	2008	[105]
3F73	dsRNA/Ago complex	*Thermus thermophilus*	X-ray	3.0 Å	2008	[105]
3HJF	RNA-DNA/Ago complex	*Thermus thermophilus*	X-ray	3.0 Å	2009	[106]
4F3T	miRNA/Ago2 complex	*Homo sapiens*	X-ray	2.2 Å	2012	[97]
4KRE	Sf9/Ago1 complex	*Homo sapiens*	X-ray	1.7 Å	2013	[107]
4N41	dsDNA/Argonaute complex	*Thermus thermophilus*	X-ray	2.2 Å	2014	[92]
4OLA	Apo form Argonaute 2	*Homo sapiens*	X-ray	2.3 Å	2014	[108]
4W5N	RNA/Argonaute 2 complex	*Homo sapiens*	X-ray	2.9 Å	2014	[109]
4Z4D	Target RNA/Ago2 complex	*Homo sapiens*	X-ray	1.6 Å	2015	[110]
5AWH	RNA-DNA/Argonaute complex	*Cereibacter sphaeroides*	X-ray	2.0 Å	2015	[111]
5JS1	siRNA/Ago2 complex	*Homo sapiens*	X-ray	2.5 Å	2016	[112]
5W6V	RNA/Ago1/GW182 complex	*Homo sapiens*	X-ray	2.8 Å	2017	[113]
5VM9	Guide RNA/Ago3 complex	*Homo sapiens*	X-ray	3.2 Å	2017	[114]
6CBD	RNA/Ago2 complex	*Homo sapiens*	X-ray	2.2 Å	2018	[115]
6D8A	RNA-DNA/Argonaute complex	*Cereibacter sphaeroides*	X-ray	2.2 Å	2018	[116]
6QZK	DNA/Argonaute complex	*Clostridium butyricum*	X-ray	3.5 Å	2019	[117]
6N4O	MiR-122/Ago2 complex	*Homo sapiens*	X-ray	2.9 Å	2019	[118]
6OON	Guide RNA/Ago4 complex	*Homo sapiens*	X-ray	1.9 Å	2019	[93]
6MFN	MiR-27a/Ago2 complex	*Homo sapiens*	X-ray	2.5 Å	2019	[119]
7KI3	MiR-122/target/Ago2	*Homo sapiens*	X-ray	3.0 Å	2021	[120]

The molecular details for the human RISC loading mechanism and target recognition by AGO2 have been extensively studied by structural methods, namely X-ray crystallography (Table 3) [108,112]. Structural studies in human AGO2 in complex with different guide and target RNAs were used to determine the rules for target recognition and binding [109]. AGO2 acts in a sequential manner during the assembly of the RISC complex. First, the guide miRNA is selected from the mature dsRNA by interaction with the Dicer enzyme and blocked by AGO2 protein, avoiding spurious interactions with other cellular RNAs. The miRNA–AGO2 complex will be initially directed to different mRNAs by the nucleotides 2 to 5 that will be used to identify potential targets. In the case of a positive selection, the remaining nucleotides of the miRNA seed sequence will be used by AGO2 to verify and engage a final mRNA target (Figure 4c–e) [109]. This “sequence-walking” process is accompanied by structural rearrangements of the PIWI and PAZ domains in AGO2 and could be complemented by an additional compensatory base pairing in the 3′-end of the miRNA [109,121].

**Figure 5:**
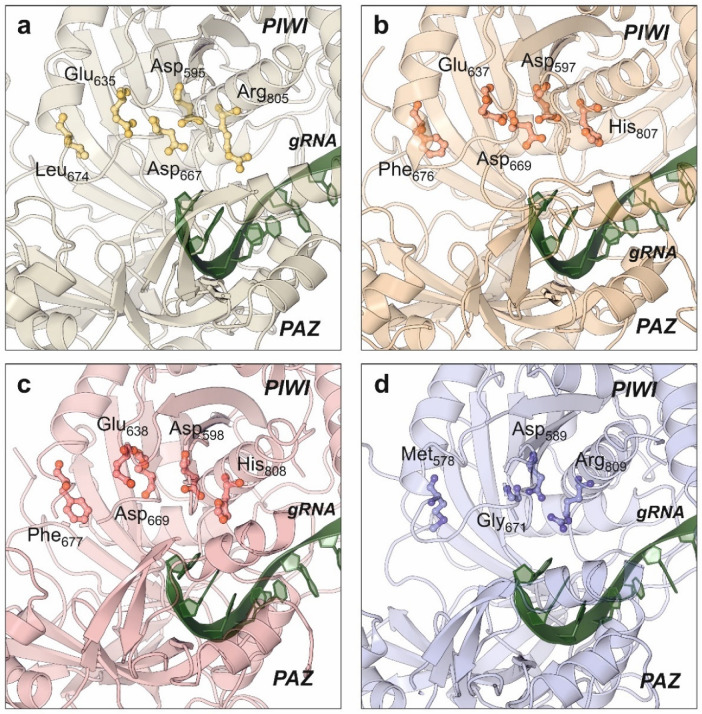
Conservation of the catalytic residues in the PIWI domain of all human Argonaute proteins as determined by X-ray crystallography. The catalytic amino acids are in the side surface of the PIWI domain, close to the RNA-binding groove. Among all the human Argonaute paralogs, only AGO2 and AGO3 retain the catalytic amino acid tetrad D-E-D-H. (**a**) AGO1 (PDB code: 4KRE), (**b**) AGO2 (PDB code: 4W5N), (**c**) AGO3 (PDB code: 5VM9), (**d**) AGO4 (PDB code: 6OON). All the figure panels were prepared with the Protein Imager software [39].

Catalytic properties of human AGO2 have been also elucidated by structural studies [108,112,118]. PIWI domain from human AGO2 showed an RNAse-like fold that remains in close contact to the substrate-binding cavity formed by the PAZ and N-terminal domains. Compared with the bacterial relatives, human AGO2 maintains the conserved catalytic aspartate pocket (D-E-D) located in the surface of the PIWI domain, with an additional histidine that stabilizes the active residues. This histidine is replaced by arginine in the bacterial Argonaute proteins [122]. Despite the initial biochemical studies that pointed AGO2 as the only human Argonaute harboring RNA-slicing activity, the structure of AGO3 also showed the presence of the catalytic residues D-E-D-H and an almost identical configuration of the enzyme active center (Figure 5) [114]. Moreover, structural studies combined with biochemical assays allowed to conclude that the substrate specificity of human AGO3 is slightly different from AGO2 [114,123]. The AGO3 substrate-binding pocket has a different conformation from the one observed in AGO2 (Figure 5b,c). Unlike AGO2, the AGO3 slicing activity depends on the establishment of additional base pairs that complement the canonical seed bonds between the miRNA and its target [123].

The structures of human AGO1 [107] and AGO4 [93] proteins have also been determined by X-ray crystallography (Figure 5a,d). Interestingly, both members of the Argonaute family do not have slicer activity, but their structures provided an important contribution to understand the miRNA regulatory action. Targeted mutation of AGO1 catalytic site substituting Leu674 (L674F) and Arg805 (R805H) to generate a canonical D-E-D-H site did not result in an activation of AGO1 slicing activity, suggesting the involvement of additional structural elements [107]. The engineering of human AGO1 to produce an enzyme harboring the RNA slicing activity also required the swapping of the N-terminal domain from AGO2, suggesting a dynamic interaction between both domains for the regulatory activity [107]. Additional data supporting the domain interactions in Argonaute proteins were obtained from the high-resolution structure of human AGO4 protein determined by X-ray crystallography [93]. The quality of the obtained data allowed to determine that the PIWI domain could be clearly divided into two subdomains whose packing is enhanced by the presence of the guide RNA, thereby rearranging the active-site residues and increasing the affinity for RISC proteins [93]. Consequently, domain interactions in human AGOs are very relevant for their intrinsic catalytical properties but also for the overall arrangement of the RISC that contributes to the final miRNA-based regulation [93,124].

In miRNA-driven regulation, AGO proteins are helped by additional partners that belong to the RISC. Among them, GW183 is the most relevant, being necessary for a proper engagement of AGO proteins into the RISC. In humans, GW183 interacts with AGO proteins via three of the GW-WG repeats in its Argonaute-binding domain as determined by X-ray crystallography [113]. Human AGO1 and AGO2 harbor single GW183-binding sites. When AGOs are loaded with a guide RNA, the affinity for GW183 increases and the AGO-GW183 complex facilitates the assembly of a stable and functional RISC [113,125].

Despite the detailed structural knowledge about individual elements of the RISC, the complete high-resolution characterization of the functional complex remains elusive. A classical paper published in 2009 by Eva Nogales’ laboratory described a low resolution structure (~30 Å) of the in vitro reconstituted human RISC determined by single particle cryo-EM [83]. Electron maps showed an L-shaped structure where it was possible to dock the structures of Dicer, TRBP and AGO2. The low resolution of the structure prevented deriving molecular details of the complex dynamics but allowed to describe the established interactions between AGO2 and Dicer that support the coupling of miRNA biogenesis and action [83].

## 5. Conclusions and Further Perspectives

The biosynthesis of miRNAs is a very peculiar biogenesis pathway when compared with other ncRNAs. The nature of the specialized nucleases that process the precursor transcriptional units until the production of mature miRNAs has been subject of detailed functional studies facilitated by the most advanced structural biology techniques during the last two decades. Structural biology has dissected the molecular details of the processing of miRNA precursors by Drosha and Dicer enzymes and the target specificity demonstrated by miRNAs over mRNAs. The post-transcriptional regulatory mechanisms mediated by miRNAs were also characterized by structural biology methods, providing molecular details for their function and actions within cells. Despite all the available structural information, some specific molecular details of miRNA biogenesis and mechanism of action remain uncharacterized. These uncharacterized details are directly related with the intrinsic structure of the processor enzymes that either contain long flexible stretches as Drosha or interact with intrinsically disordered partners. Another point that would deserve deeper characterization is the structure and dynamics of the multi-protein complexes involved either in miRNA processing or regulatory activity namely the RISC. The currently available low-resolution Cryo-EM data obtained from the in vitro reconstituted RISC does not provide sufficient details for understanding the complex dynamics involved in target recognition and regulation. In consequence, a more detailed characterization of the RISC would be required to complete all the molecular details of miRNA action.

Connecting with the existence of intrinsically unfolded proteins associated with the maturation miRNA machinery, dynamic associations of protein partners that can modulate the miRNA biogenesis under different conditions are expected to take place. This fact can be clearly illustrated by the recent characterization of the ERH splicing factor as a helping partner in the maturation of clustered miRNAs by the Drosha/DGCR8 complex, partially explaining the different expression levels observed in miRNAs whose precursors are in tandem clusters. Dynamic complexes will be more difficult to characterize by structural methods, requiring sophisticated protocols that combine different techniques such as mass spectrometry, time-resolved structural biology and classical molecular biology.

Finally, we could not disregard the contribution of computer methods to the structural biology with potential implications in the miRNA field. For instance, molecular dynamics simulations have been recently applied to the characterization of AGO2 interactions with miRNAs and mRNA targets, showing specific details involving structural elements present in the targeted mRNA as relevant characteristics to consider miRNA action [126,127]. Other computer methods such as AI-driven protein folding predictions are also very promising tools to understand dynamic structural phenomena which are difficult to tackle by wet-lab protocols. These methods alone or in combination to the experimental data available could be applied to the study of the dynamics of nuclear and cytoplasmic processor complexes and the RISC.

## Figures and Tables

**Table 1:** PDB database entries describing total or partial structures from components of the nuclear microprocessor for miRNA maturation.

PDB Code	Biomolecule	Species	Technique	Resolution	Year	Reference
1X47	dsRNA binding domain of DGCR8	*Homo sapiens*	NMR	N/A	2005	Unpublished
2YT4	DGCR8 core	*Homo sapiens*	X-ray	2.6 Å	2007	[32]
2KHX	dsRNA binding domain of Drosha	*Homo sapiens*	NMR	N/A	2010	[35]
3LE4	DGCR8 dimerization domain	*Homo sapiens*	X-ray	1.7 Å	2010	[33]
4ER5	DGCR8 dimerization domain	*Xenopus laevis*	X-ray	1.9 Å	2013	[34]
5B16	Drosha–DGCR8 complex	*Homo sapiens*	X-ray	3.2 Å	2016	[36]
6V5B	Drosha–DGCR8–pri-miRNA complex	*Homo sapiens*	Cryo-EM	3.7 Å	2020	[37]
6V5C	Drosha–DGCR8–pri-miRNA complex	*Homo sapiens*	Cryo-EM	4.4 Å	2020	[37]
6XLE	Drosha–DGCR8 complex	*Homo sapiens*	Cryo-EM	4.2 Å	2020	[38]
6XLD	Pri-miRNA bound to Drosha-DGCR8	*Homo sapiens*	Cryo-EM	3.9 Å	2020	[38]
7CNC	ERH in complex with DGCR8	*Homo sapiens*	X-ray	1.6 Å	2020	[28]

## Supplementary Materials

The following supplementary materials are available online at https://www.mdpi.com/article/10.3390/ncrna8010010/s1, Figure S1: Structure-based sequence alignment of the human AGO1, AGO2, AGO3 and AGO4 proteins, showing the conserved segments, the consensus sequence and the catalytic triad DEDH present in AGO2 and AGO3 proteins; Figure S2: Structural analysis of human AGO paralogs with experimental structure information showing a structural superposition of the four atomic models with the binding position of the guiding RNA strand (panel **a**) and the representation of the electrostatic surface potential in AGO2 structure to highlight the RNA-binding pocket (panel **b**).
