# Supplementary material for: A Structural View of miRNA Biogenesis and Function
Source: Noncoding RNA. 2022 Jan 18;8(1):10. doi: 10.3390/ncrna8010010 (PMC8874510; doi:10.3390/ncrna8010010)
Supplement: Supplementary file 1 [file ncrna-08-00010-s001.zip › Figure S1.pdf]

```
Conservation:       9555 599  5555555555   5  95995 99 955959599 9 5 99999595959999 95999
AGO1           1    M---EAGPSGAAAGAYLPPLQQVFQAPRRPGIGTVGKPIKLLANYFEVDIPKIDVYHYEVDIKPDKCPRR   67
AGO4           1    M--EALGPGPP---------ASLFQPPRRPGLGTVGKPIRLLANHFQVQIPKIDVYHYDVDIKPEKRPRR   59
AGO3           1    MEIGSAGPAGA---------QPLLMVPRRPGYGTMGKPIKLLANCFQVEIPKIDVYLYEVDIKPDKCPRR   61
AGO2           1    M-YSGAGPALAPPAPPPPIQGYAFKPPPRPDFGTSGRTIKLQANFFEMDIPKIDIYHYELDIKPEKCPRR   69
Consensus aa:       M....hGPt.s..........hhbsP.RPshGT.G+sI+LbANhFphpIPKIDlYhY-lDIKP-K.PRR
Consensus ss:                           hh               eeeeeeeeeeeee    eeeeeeeee     hh

Conservation:       999959  995999 599999 955995 5 995 59955   95 599599959995 999955555 9
AGO1          68    VNREVVEYMVQHFKPQIFGDRKPVYDGKKNIYTVTALPIGNERVDFEVTIPGE-GKDRIFKVSIKWLAIV  136
AGO4          60    VNREVVDTMVRHFKMQIFGDRQPGYDGKRNMYTAHPLPIGRDRVDMEVTLPGE-GKDQTFKVSVQWVSVV  128
AGO3          62    VNREVVDSMVQHFKVTIFGDRRPVYDGKRSLYTANPLPVATTGVDLDVTLPGEGGKDRPFKVSIKFVSRV  131
AGO2          70    VNREIVEHMVQHFKTQIFGDRKPVFDGRKNLYTAMPLPIGRDKVELEVTLPGE-GKDRIFKVSIKWVSCV  138
Consensus aa:       VNRElV-.MVpHFK.pIFGDRpPs@DG++shYTh.sLPltpp.V-h-VTlPGE.GKDp.FKVSlp@lt.V
Consensus ss:       hhhhhhhhhhhhhhhhh    eeeee hhheee         eeeeeeee        eeeeeeeee  ee

Conservation:       9   95559 5    59 555555555 559599  99959959999999995 5995 59999999999
AGO1         137    SWRMLHEALVSGQIPVPLE--------SVQALDVAMRHLASMRYTPVGRSFFSPPEGYYHPLGGGREVWF  198
AGO4         129    SLQLLLEALAGHLNEVPDD--------SVQALDVITRHLPSMRYTPVGRSFFSPPEGYYHPLGGGREVWF  190
AGO3         132    SWHLLHEVLTGRTLPEPLELDKPISTNPVHAVDVVLRHLPSMKYTPVGRSFFSAPEGYDHPLGGGREVWF  201
AGO2         139    SLQALHDALSGRLPSVPFE--------TIQALDVVMRHLPSMRYTPVGRSFFTASEGCSNPLGGGREVWF  200
Consensus aa:       ShphLh-hLst.....P.-........slpAlDVhhRHLsSM+YTPVGRSFFossEGh.pPLGGGREVWF
Consensus ss:       ehhhhhhhh        hh         hhhhhhhhhhh     eeee           ee      eeeee

Conservation:       99999999555959999999999999959999595959999995 59  99  5999995995999995999959
AGO1         199    GFHQSVRPAMWKMMLNIDVSATAFYKAQPVIEFMCEVLDIRNIDEQPKPLTDSQRVRFTKEIKGLKVEVT  268
AGO4         191    GFHQSVRPAMWNMMLNIDVSATAFYRAQPIIEFMCEVLDIQNINEQTKPLTDSQRVKFTKEIRGLKVEVT  260
AGO3         202    GFHQSVRPAMWKMMLNIDVSATAFYKAQPVIQFMCEVLDIHNIDEQPRPLTDSHRVKFTKEIKGLKVEVT  271
AGO2         201    GFHQSVRPSLWKMMLNIDVSATAFYKAQPVIEFVCEVLDFKSIEEQQKPLTDSQRVKFTKEIKGLKVEIT  270
Consensus aa:       GFHQSVRPthWpMMLNIDVSATAFY+AQPlIpFhCEVLDhpsIpEQ.+PLTDSpRV+FTKEI+GLKVElT
Consensus ss:        eeeeeeee   eeeeeeeeeeeee    hhhhhhhhh             hhhhhhhhhhh   eeeee

Conservation:       9995959999999999999999999999959 9955959999995 55 9595999999999999999999
AGO1         269    HCGQMKRKYRVCNVTRRPASHQTFPLQLESGQTVECTVAQYFKQKYNLQLKYPHLPCLQVGQEQKHTYLP  338
AGO4         261    HCGQMKRKYRVCNVTRRPASHQTFPLQLENGQAMECTVAQYFKQKYSLQLKYPHLPCLQVGQEQKHTYLP  330
AGO3         272    HCGTMRRKYRVCNVTRRPASHQTFPLQLENGQTVERTVAQYFREKYTLQLKYPHLPCLQVGQEQKHTYLP  341
AGO2         271    HCGQMKRKYRVCNVTRRPASHQTFPLQQESGQTVECTVAQYFKDRHKLVLRYPHLPCLQVGQEQKHTYLP  340
Consensus aa:       HCGpM+RKYRVCNVTRRPASHQTFPLQbEsGQhhE.TVAQYF+p+@pL.L+YPHLPCLQVGQEQKHTYLP
Consensus ss:              eeee           eeeee       eeeehhhhhhh   eee     eeee      eeee

Conservation:       9999999999999999999999999959999999999999959  555 55   995   9955 9 5595 5
AGO1         339    LEVCNIVAGQRCIKKLTDNQTSTMIKATARSAPDRQEEISRLMKNASY--NLDPYIQEFGIKVKDDMTEV  406
AGO4         331    LEVCNIVAGQRCIKKLTDNQTSTMIKATARSAPDRQEEISRLVKSNSMVGGPDPYLKEFGIVVHNEMTEL  400
AGO3         342    LEVCNIVAGQRCIKKLTDNQTSTMIKATARSAPDRQEEISRLVRSANY--ETDPFVQEFQFKVRDEMAHV  409
AGO2         341    LEVCNIVAGQRCIKKLTDNQTSTMIRATARSAPDRQEEISKLMRSASF--NTDPYVREFGIMVKDEMTDV  408
Consensus aa:       LEVCNIVAGQRCIKKLTDNQTSTMI+ATARSAPDRQEEIS+Lh+sssh....DP@lpEF.h.V+s-Mhcl
Consensus ss:       eeeeeee          hhhhhhhhhhhh   hhhhhhhhhhhhh      hhhhhhh   ee   eeee

Conservation:       99999559 5599999   999 59999995999  9599959959999959 9959  99 995999999
AGO1         407    TGRVLPAPILQYGGRNRAIATPNQGVWDMRGKQFYNGIEIKVWAIACFAPQKQCREEVLKNFTDQLRKIS  476
AGO4         401    TGRVLPAPMLQYGGRNKTVATPNQGVWDMRGKQFYAGIEIKVWAVACFAPQKQCREDLLKSFTDQLRKIS  470
AGO3         410    TGRVLPAPMLQYGGRNRTVATPSHGVWDMRGKQFHTGVEIKMWAIACFATQRQCREEILKGFTDQLRKIS  479
AGO2         409    TGRVLQPPSILYGGRNKAIATPVQGVWDMRNKQFHTGIEIKVWAIACFAPQRQCTEVHLKSFTEQLRKIS  478
Consensus aa:       TGRVL.sP.lbYGGRN+hlATPspGVWDMRsKQF@sGlEIKhWAlACFAsQ+QCpE.hLKsFT-QLRKIS
Consensus ss:       eeee    eeee    eee      eee     ee      eeeeeee       hhhhhhhhhhhhhhh

Conservation:       5999999999999999999999999995999599 99995 9999999999999999995999999999
AGO1         477    KDAGMPIQGQPCFCKYAQGADSVEPMFRHLKNTYSGLQLIIVILPGKTPVYAEVKRVGDTLLGMATQCVQ  546
AGO4         471    KDAGMPIQGQPCFCKYAQGADSVEPMFKHLKMTYVGLQLIVVILPGKTPVYAEVKRVGDTLLGMATQCVQ  540
AGO3         480    KDAGMPIQGQPCFCKYAQGADSVEPMFRHLKNTYSGLQLIIVILPGKTPVYAEVKRVGDTLLGMATQCVQ  549
AGO2         479    RDAGMPIQGQPCFCKYAQGADSVEPMFRHLKNTYAGLQLVVVILPGKTPVYAEVKRVGDTVLGMATQCVQ  548
Consensus aa:       +DAGMPIQGQPCFCKYAQGADSVEPMF+HLK.TYsGLQLllVILPGKTPVYAEVKRVGDTlLGMATQCVQ
Consensus ss:       hh         eeee    hhhhhhhhhhhhhh    eeeeeee     hhhhhhhhhhh     eeeee
```

```
Conservation:            5999 5959999999999999995999959959595595 999999999999999999999999995999999
AGO1                547  VKNVVKTSPQTLSNLCLKINVKLGGINNILVPHQRSAVFQQPVIFLGADVTHPPAGDGKKPSITAVVGSM  616
AGO4                541  VKNVVKTSPQTLSNLCLKINAKLGGINNVLVPHQRPSVFQQPVIFLGADVTHPPAGDGKKPSIAAVVGSM  610
AGO3                550  VKNVIKTSPQTLSNLCLKINVKLGGINNILVPHQRPSVFQQPVIFLGADVTHPPAGDGKKPSIAAVVGSM  619
AGO2                549  MKNVQRTTPQTLSNLCLKINVKLGGVNNILLPQGRPPVFQQPVIFLGADVTHPPAGDGKKPSIAAVVGSM  618
Consensus aa:            hKNV.+ToPQTLSNLCLKINhKLGGlNNlLlPp.RssVFQQPVIFLGADVTHPPAGDGKKPSIhAVVGSM
Consensus ss:             hh     hhhhhhhhhhhhhhh     ee            eeeeeeeee          eeeeeeeee

Conservation:            95995999999999  999955555555559599  9999999999999999999995995995999  9
AGO1                617  DAHPSRYCATVRVQRPRQEI----------IEDLSYMVRELLIQFYKSTRFKPTRIIFYRDGVPEGQLPQ  676
AGO4                611  DGHPSRYCATVRVQTSRQEISQELLYSQEVIQDLTNMVRELLIQFYKSTRFKPTRIIYYRGGVSEGQMKQ  680
AGO3                620  DAHPSRYCATVRVQRPRQEI----------IQDLASMVRELLIQFYKSTRFKPTRIIFYRDGVSEGQFRQ  679
AGO2                619  DAHPNRYCATVRVQQHRQEI----------IQDLAAMVRELLIQFYKSTRFKPTRIIFYRDGVSEGQFQQ  678
Consensus aa:            DtHPsRYCATVRVQp.RQEI..........IpDLs.MVRELLIQFYKSTRFKPTRII@YRsGVsEGQh.Q
Consensus ss:                 eeeeeeeee     h          hhhhhhhhhhhhhhhhhhh   hheeeeee     hhhhh

Conservation:            55  995999 999 995995999959999999999999595 99595999599999995 9999 9999
AGO1                677  ILHYELLAIRDACIKLEKDYQPGITYIVVQKRHHTRLFCADKNERIGKSGNIPAGTTVDTNITHPFEFDF  746
AGO4                681  VAWPELIAIRKACISLEEDYRPGITYIVVQKRHHTRLFCADKTERVGKSGNVPAGTTVDSTITHPSEFDF  750
AGO3                680  VLYYELLAIREACISLEKDYQPGITYIVVQKRHHTRLFCADRTERVGRSGNIPAGTTVDTDITHPYEFDF  749
AGO2                679  VLHHELLAIREACIKLEKDYQPGITFIVVQKRHHTRLFCTDKNERVGKSGNIPAGTTVDTKITHPTEFDF  748
Consensus aa:            lh@.ELlAIRcACIpLEcDYpPGIT@IVVQKRHHTRLFChD+sERlG+SGNlPAGTTVDopITHP.EFDF
Consensus ss:            hhhhhhhhhhhhhhhh       eeeeeee     eeee                eee   ee      e

Conservation:            99999999999999999 999999 9559999 9999999999999999999999999 99999999999
AGO1                747  YLCSHAGIQGTSRPSHYYVLWDDNRFTADELQILTYQLCHTYVRCTRSVSIPAPAYYARLVAFRARYHLV  816
AGO4                751  YLCSHAGIQGTSRPSHYQVLWDDNCFTADELQLLTYQLCHTYVRCTRSVSIPAPAYYARLVAFRARYHLV  820
AGO3                750  YLCSHAGIQGTSRPSHYHVLWDDNCFTADELQLLTYQLCHTYVRCTRSVSIPAPAYYAHLVAFRARYHLV  819
AGO2                749  YLCSHAGIQGTSRPSHYHVLWDDNRFSSDELQILTYQLCHTYVRCTRSVSIPAPAYYAHLVAFRARYHLV  818
Consensus aa:            YLCSHAGIQGTSRPSHY.VLWDDN.FotDELQlLTYQLCHTYVRCTRSVSIPAPAYYA+LVAFRARYHLV
Consensus ss:            eee            eeeeee      hhhhhhhhhhhhhheee        hhhhhhhhhhhhhhhhh

Conservation:            99599959999 99999999599999999 95995599999
AGO1                817  DKEHDSGEGSHISGQSNGRDPQALAKAVQVHQDTLRTMYFA  857
AGO4                821  DKDHDSAEGSHVSGQSNGRDPQALAKAVQIHHDTQHTMYFA  861
AGO3                820  DKEHDSAEGSHVSGQSNGRDPQALAKAVQIHQDTLRTMYFA  860
AGO2                819  DKEHDSAEGSHTSGQSNGRDHQALAKAVQVHQDTLRTMYFA  859
Consensus aa:            DK-HDStEGSHhSGQSNGRD.QALAKAVQlHpDTb+TMYFA
Consensus ss:                                           hhhhhh
```
