# Supplementary figures and images for: A Structural View of miRNA Biogenesis and Function
Source: Noncoding RNA. 2022 Jan 18;8(1):10. doi: 10.3390/ncrna8010010 (PMC8874510; doi:10.3390/ncrna8010010)

**a**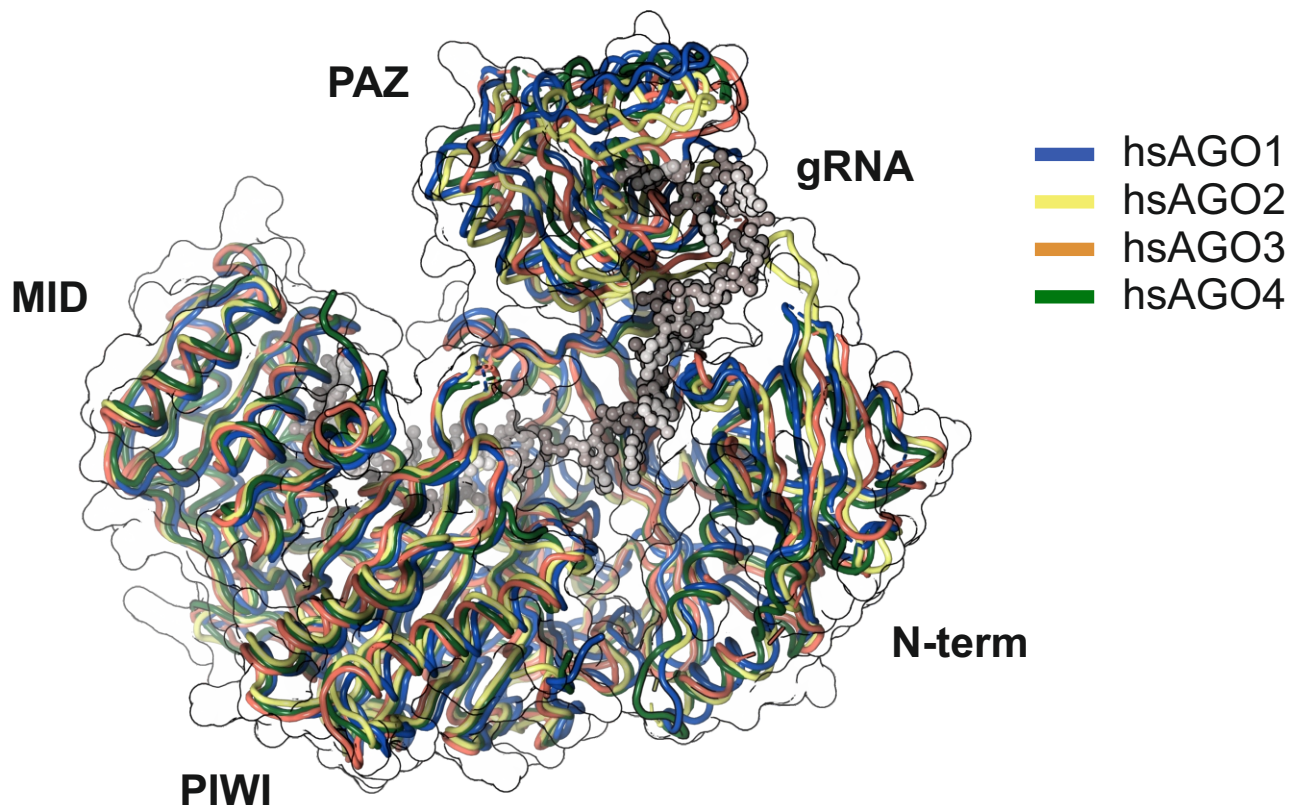**b**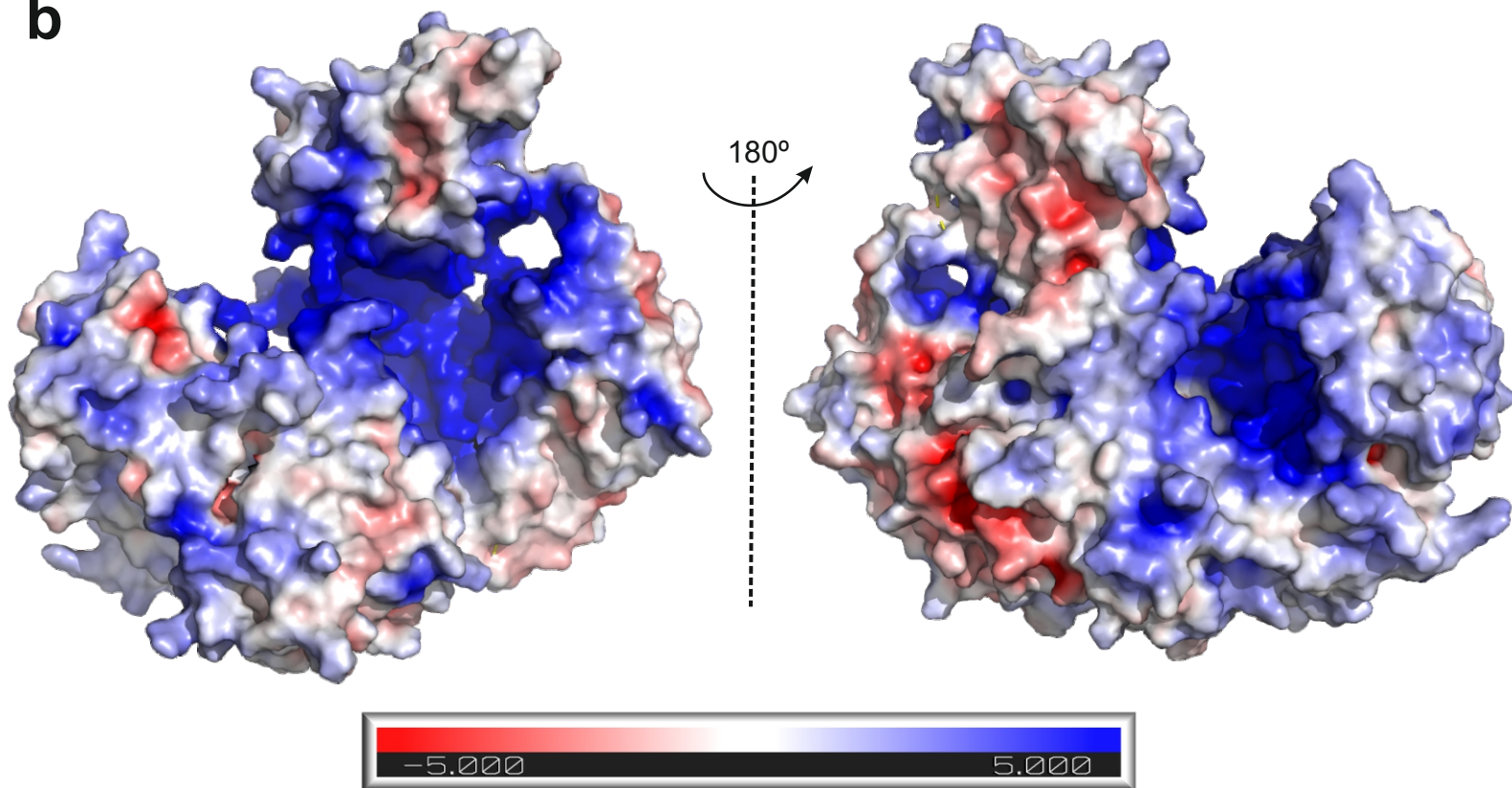

Supplement: Supplementary file 1 [file ncrna-08-00010-s001.zip › Figure S2.pdf]
